# Synthesis and Antimicrobial Activity of Some New Thiadiazoles, Thioamides, 5-Arylazothiazoles and Pyrimido[4,5-*d*][1,2,4]triazolo[4,3-*a*]pyrimidines

**DOI:** 10.3390/molecules21081072

**Published:** 2016-08-17

**Authors:** Abdou O. Abdelhamid, Ibrahim E. El Sayed, Mohamed Z. Hussein, Mangoud M. Mangoud

**Affiliations:** 1 Department of Chemistry, Faculty of Science, Cairo University, Giza 12613, Egypt; 2 Department of Chemistry, Faculty of Science, El Menoufia University, Shebin El Koom 32511, Egypt; ibrahimtantawy@yahoo.co.uk; 3 Environmental Research Department, National Center for Social and Criminological Research, Ibn Khaldoun Square, Mohandesin, Zamalek, Giza 11561, Egypt; mohamedzaki1955@yahoo.com (M.Z.H.); mangoud_online@yahoo.com (M.M.M.)

**Keywords:** hydrazonoyl halides, thioamides, 1,3,4-thiadiazoles, thiazoles, pyrimido[4,5-*d*][1,2,4]triazolo[4,3-*a*]pyrimidines, antimicrobials

## Abstract

A novel series of 1,3,4-thiadiazoles, 5-arylazothiazoles and hexahydropyrimido-[4,5-*d*][1,2,4]triazolo[4,3-*a*]pyrimidines were synthesized via reaction of hydrazonoyl halides with each of alkyl carbothioates, carbothioamides and 7-thioxo-5,6,7,8-tetrahydropyrimido-[4,5-*d*]pyrimidine-2,4(1*H*,3*H*)-diones in the presence of triethylamine. The structures of the newly synthesized compounds were established based on their spectral data, elemental analyses and alternative synthetic routes whenever possible. Also, the newly synthesized compounds were screened for their antimicrobial activity against various microorganisms.

## 1. Introduction

Hydrazonoyl halides have been widely used as reagents for the synthesis of heterocyclic compounds, both through condensation reactions, and as precursors of nitrilimines, which can undergo cycloaddition with dipolarophiles [1,2]. 1,3,4-Thiadiazoles are among the most common heterocyclic pharmacophores. They display a broad spectrum of biological activities, including antimicrobial [3] anticancer [4,5], antioxidant [6], antidepressant [7], anticonvulsant [8,9], and antihypertensive activity [10] as well as acetylcholinesterase inhibition for the treatment of Alzheimer disease [11,12]. In addition, thiazoles can found in drugs developed for the treatment of allergies [13], hypertension [14], inflammation [15], schizophrenia [16], bacterial infections [17], HIV [18], sleep disorders [19] and more recently, for the treatment of pain [20], as fibrinogen receptor antagonists with antithrombotic activity [21], and as new inhibitors of bacterial DNA gyrase B [22]. 1,2,4-Triazolopyrimidines have also attracted growing interest due to their important pharmacological activities, such as antitumor, antimalarial, antimicrobial, anti-inflammatory, antifungal properties, and their potency in macrophage activation [23,24,25,26,27].

## 2. Results and Discussion

**Scheme 1:**
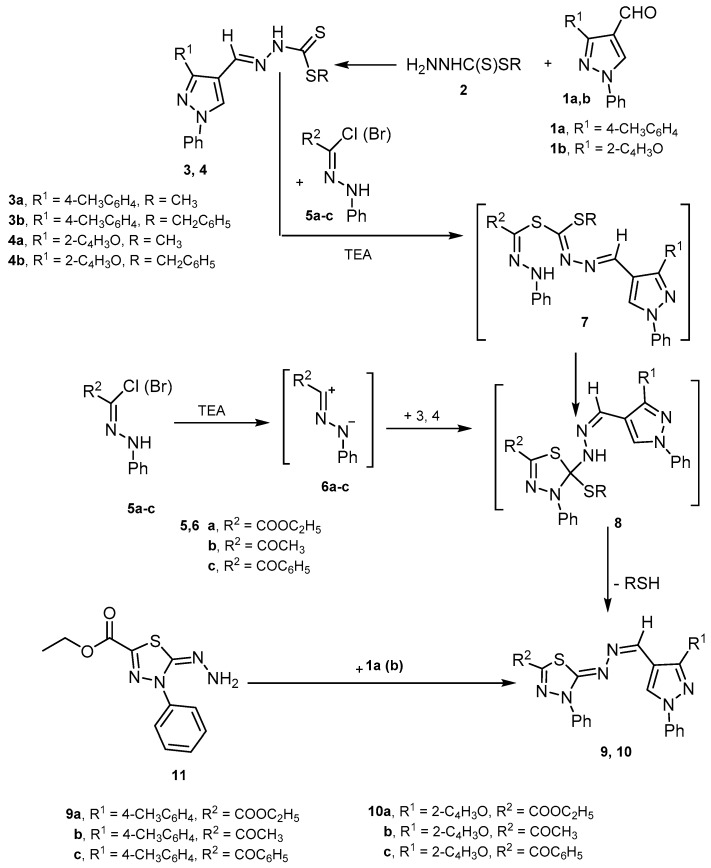
Synthesis of 1,3,4-thiadiazole derivatives **9a**–**c** and **10a**–**c**.

Treatment of methyl 2-methylenehydrazinecarbodithioates **3a** and [28] **4a** with ethyl 2-chloro-2-(2-phenylhydrazono)acetate (**5a**) in ethanol containing triethylamine gave ethyl 5-({[1*H*-pyrazol-4-yl]methylene}hydrazono)-1,3,4-thiadiazole-2-caboxylates **9a** and **10a**, respectively (Scheme 1). The structure **9a** was established by elemental analysis, ^1^H-NMR, IR spectroscopy, mass spectrometry and alternative syntheses. Thus, (5*E*)-ethyl 5-hydrazono-4,5-dihydro-4-phenyl-1,3,4-thiadiazole-2-carboxylate [29] (**11**) was reacted with **1a** and **1b** to give products identical in all aspects (m.p., mixed m.p. and spectra) with **9a** and **10a**, respectively. In addition, benzyl 2-((1-phenyl-3-(*p*-tolyl)-1*H*-pyrazol-4-yl)-methylene)-hydrazine-1-carbodithioate (**3b**) was reacted with **5a** in ethanolic triethylamine to give a product identical in all aspects (m.p., mixed m.p. and spectra) with **9a**. In light of the foregoing results, the mechanism outlined in Scheme 1 seems to be the most plausible pathway for the formation of **9** from the reaction of the **5** with **3a** or **3b**. The reaction involves initial formation of thiohydrazonate **7**, which undergoes intermolecular cyclization directly to yield intermediate **8** or via 1,3-dipolar cycloaddition of nitrilimine **6** (generated in situ from **5** with triethylamine) to the C=S of **3** or **4** to yield intermediate **8**. Formations of **7** and **8** are similar to the reaction of hydrazonoyl chloride with 1-phenyl-1,4-dihydrotetrazole-5-thione [30] and 5-phenyl-1,3,4-thiadiazole-2(3*H*)-thione [31]. Intermediate **8** was converted to **9** by elimination of alkyl mercaptan. Analogously, **5b**, **5c** were reacted separately with **3a**, **3b**, **4a** or **4b** in ethanolic triethyamine to afford 2,3-dihydro-1,3,4-thiadiazoles **9b**–**c** and **10a**–**c**, respectively (Scheme 1).

**Scheme 2:**
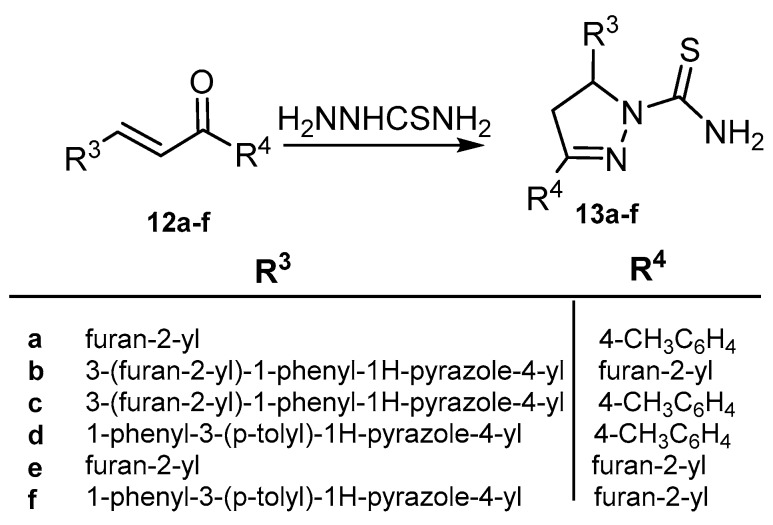
Synthesis of thioamide derivatives **13a**–**f**.

Treatment of 3-(furan-2-yl)-1-(*p*-tolyl)prop-2-en-1-one (**12a**), 1-(furan-2-yl)-3-(3-(furan-2-yl)-1-phenyl-1*H*-pyrazol-4-yl)prop-2-en-1-one (**12b**), 3-(3-(furan-2-yl)-1-phenyl-1*H*-pyrazol-4-yl)-1-(*p*-tolyl)prop-2-en-1-one (**12c**), 3-(1-phenyl-3-(*p*-tolyl)-1*H*-pyrazol-4-yl)-1-(*p*-tolyl)prop-2-en-1-one (**12d**), 1,3-di(furan-2-yl)prop-2-en-1-one (**12e**) and 1-(furan-2-yl)-3-(1-phenyl-3-(*p*-tolyl)-1*H*-pyrazol-4-yl)-prop-2-en-1-one (**12f**) with thiosemicarbazide in boiling ethanol containing potassium hydroxide gave 5-(furan-2-yl)-3-(*p*-tolyl)-4,5-dihydro-1*H*-pyrazole-1-carbothioamide (**13a**), 3’,5-di(furan-2-yl)-1’-phenyl-3,4-dihydro-1’*H*,2*H*-[3,4’-bipyrazole]-2-carbothioamide (**13b**), 3’-(furan-2-yl)-1’-phenyl-5-(*p*-tolyl)-3,4-dihydro-1’*H*,2*H*-[3,4’-bipyrazole]-2-carbothioamide (**13c**), 1’-phenyl-3’,5-di-*p*-tolyl-3,4-dihydro-1’*H*,2*H*-[3,4’-bipyrazole]-2-carbothioamide (**13d**), 3,5-di(furan-2-yl)-4,5-dihydro-1*H*-pyrazole-1-carbothioamide (**13e**) and 5-(furan-2-yl)-1’-phenyl-3’-(*p*-tolyl)-3,4-dihydro-1’*H*,2*H*-[3,4’-bipyrazole]-2-carbothioamide (**13f**), respectively (Scheme 2).

**Scheme 3:**
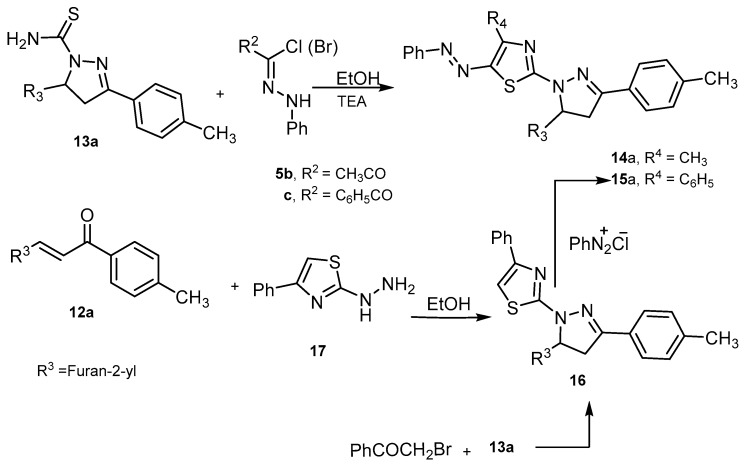
2-(5-(Furan-2-yl)-3-(*p*-tolyl)-4,5-dihydro-1*H*-pyrazol-1-yl)-4-substituted-5-(phenyldiazenyl)thiazole **14a** and **15a**.

Thus, thioamide **13a** was reacted with the hydrazonoyl halides **5b** and **5c** in boiling ethanol containing triethylamine to give 2-(5-(furan-2-yl)-3-(*p*-tolyl)-4,5-dihydro-1*H*-pyrazol-1-yl)-4-methyl-5-(phenyldiazenyl)thiazole (**14a**) and 2-(5-(furan-2-yl)-3-(*p*-tolyl)-4,5-dihydro-1*H*-pyrazol-1-yl)-4-phenyl-5-(phenyldiazenyl)thiazole (**15a**), respectively (Scheme 3). Thus, benzenediazonium chloride was reacted with 2-(5-(furan-2-yl)-3-(*p*-tolyl)-4,5-dihydro-1*H*-pyrazol-1-yl)-4-phenyl-thiazole (**16**), which was prepared via the reaction of **12a** with 2-hydrazinyl-4-phenylthiazole [32] (**17**) or the reaction of compound **13a** with ω-bromoacetophenone in pyridine at 0–5 °C to give a product identical in all aspects (mp., mixed mp. and spectra) with **15a** (Scheme 3).

**Scheme 4:**
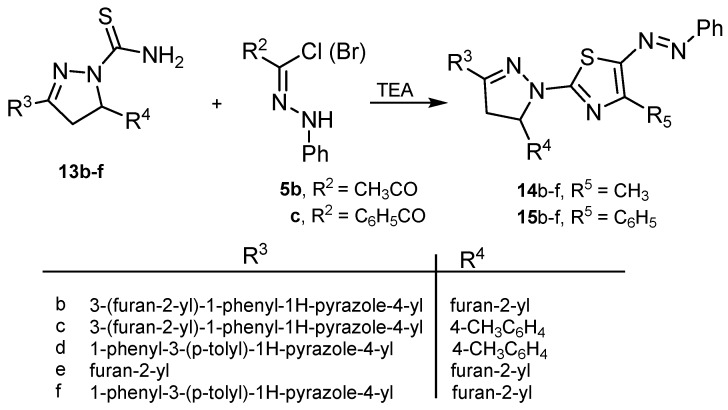
4,5-Dihydro-1*H*-pyrazol-1-yl)-4-phenyl-5-(phenyldiazenyl)thiazole derivatives **14b–f** and **15b–f**.

Analogously, treatment of the appropriate **13b**–**f** with the hydrazonoyl halides **5b** and **5c** in boiling ethanol containing triethylamine afforded 2-substituted-4,5-dihydro-1*H*-pyrazol-1-yl)-4-subsituted-5-(phenyldiazenyl)thiazoles **14b**–**f** and **15b**–**f**, respectively (Scheme 4).

**Scheme 5:**
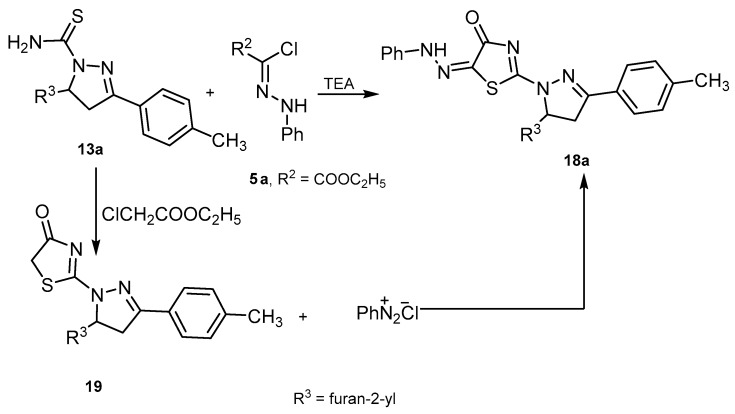
Synthesis of 2-(5-(furan-2-yl)-3-(*p*-tolyl)-4,5-dihydro-1*H*-pyrazol-1-yl)-5-(2-phenyl-hydrazono)thiazol-4(5*H*)-one **18a**.

On the other hand, treatment of ethyl 2-chloro-2-(2-phenylhydrazono)acetate (**5a**) with 5-(furan-2-yl)-3-(*p*-tolyl)-4,5-dihydro-1*H*-pyrazole-1-carbothioamide (**13a**), in boiling ethanol containing triethylamine gave one isolable product according to TLC and proved to be 2-(5-(furan-2-yl)-3-(*p*-tolyl)-4,5-dihydro-1*H*-pyrazol-1-yl)-5-(2-phenylhydrazono)thiazol-4(5*H*)-one (**18a**). Also, benzenediazonium was reacted with 2-(5-(furan-2-yl)-3-(*p*-tolyl)-4,5-dihydro-1*H*-pyrazol-1-yl)thiazol-5(4*H*)-one (**19**), which was prepared via the reaction of ethyl chloroacetate with **13a**, in ethanolic sodium acetate solution at 0–5 °C to afford a product identical in all aspects (mp, mixed mp and spectra) with **18a** (Scheme 5)

**Scheme 6:**
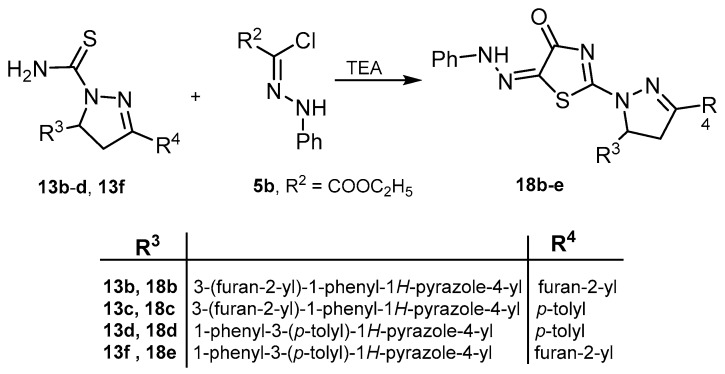
Synthesis of 3,5-substituted 4,5-dihydro-1*H*-pyrazol-1-yl)-5-(2-phenylhydrazono)thiazol-4(5*H*)-one **18b**–**e**.

Similarly, carbothioamides **13b**–**d**, and **13f** were reacted with *C*-ethoxycarbonyl-*N*-phenylhydrazonyl chloride in ethanolic triethylamine to give the corresponding thiazolone derivatives **18b**–**e**, respectively (Scheme 6).

**Scheme 7:**
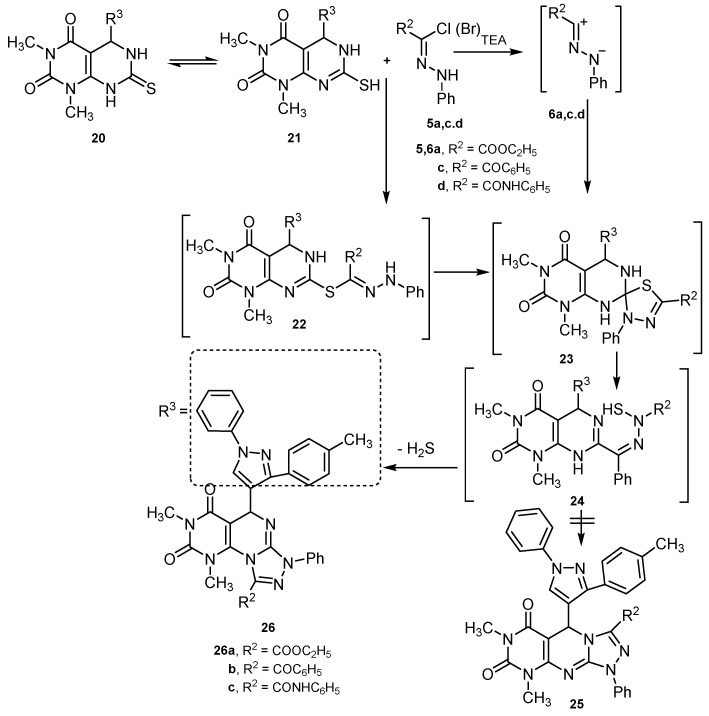
Synthesis of 1,3-dimethyl-7-phenyl-5-(1-phenyl-3-(*p*-tolyl)-1*H*-pyrazol-4-yl)-9-substituted 5,7-dihydropyrimido[5,4-*e*][1,2,4]triazolo[4,3-*a*]pyrimidine-2,4(1*H*,3*H*)-dione **26a**–**c**.

In further experiments treatment of **5a** with 5,6,7,8-tetrahydro-1,3-dimethyl-5-(1-phenyl-3-*p*-tolyl-1*H*-pyrazol-4-yl)-7-thioxopyrimido[4,5-*d*]pyrimidine-2,4(1*H*,3*H*)-dione **20** in boiling chloroform containing triethylamine produced ethyl 2,4-dioxo-1,2,3,4,5,7-hexahydro-pyrimido[5,4-*e*]-[1,2,4]-triazolo[4,3-*a*]pyrimidine-9-carboxylate **26a** in good yields (Scheme 7).

The ^1^H-NMR spectrum of **26a** showed a triplet at δ 1.18 due to methyl group of ethyl ester, a singlet at δ 2.33 of tolyl methyl group, a singlet at δ 3.33 from the two pyrimidinedione methyl groups, a quartet at δ 4.13 due to the methylene of ethyl ester, a singlet at δ 5.62 that corresponding to the pyrimidine H-4 and a multiplet at δ 6.90–7.83 that account for 14 aromatic protons and H-5 of pyrazole. In the IR spectrum, an absorption peak at 1650 cm^−1^ corresponding to conjugated carbonyl groups and an absorption near at 1615 cm^−1^ could account for the presence of the imino group.

The mechanism outlined in scheme 7 seems to be the most plausible pathway for the formation of **26a** from the reaction of **5** with **20**: (1) 1,3-addition of the thiol tautomer **21** to the nitrilimine **6a** would give the thiohydrazonate ester **22** which could undergo nucleophilic cyclization to yield spiro compounds **23**. The latter ring could then open and cyclize to yield **26a** with loss of hydrogen sulfide; and (2) 1,3-cycloaddition of nitrilimine **6a** to C=S of **20** would directly yield **22** (cf., Scheme 7). Attempts to isolate the thiohydrazonate ester **22** or intermediates **23** and **24** did not succeed even under mild conditions as they readily undergo in situ cyclization followed by elimination of hydrogen sulfide to give the final product **26** in Scheme 7.

**Scheme 8:**
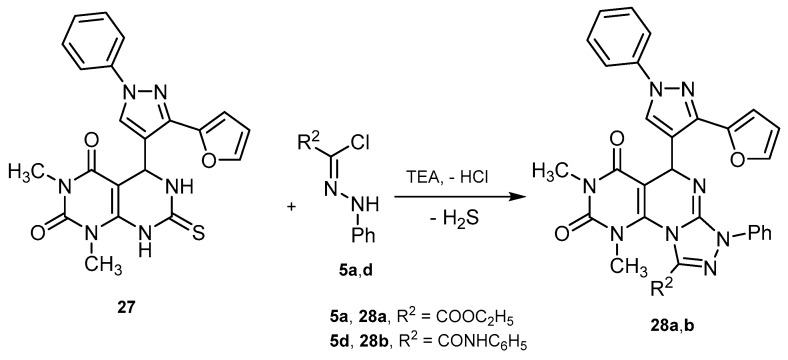
Synthesis of 5-(3-(furan-2-yl)-1-phenyl-1*H*-pyrazol-4-yl)-1,3-dimethyl-2,4-dioxo-7-phenyl-9-substituted-1,2,3,4,5,7-hexahydropyrimido[5,4-*e*][1,2,4]triazolo-[4,3-*a*]pyrimidines **28a** and **28b**.

Analogously, reaction of **20** with each of **5b** and **5d** afforded 3-acetyl-7,9-dimethyl-1-phenyl-5-(1-phenyl-3-(*p*-tolyl)-1*H*-pyrazol-4-yl)-5,9-dihydropyrimido[4,5-*d*][1,2,4]triazolo[4,3-*a*]pyrimidine-6,8(1*H*,7*H*)-dione (**26b**) and 7,9-dimethyl-6,8-dioxo-*N*,1-diphenyl-5-(1-phenyl-3-(*p*-tolyl)-1*H*-pyrazol-4-yl)-1,5,6,7,8,9-hexahydropyrimido[4,5-*d*][1,2,4]triazolo[4,3-*a*]pyrimidine-3-carboxamide (**26c**), respectively. Based on the above results, structure **25** was ruled out. This structural assignment was also consistent with literature reports which indicated that reaction of hydrazonoyl halides with 2-thioxopyrimidin-4-one yielded regioselectively the corresponding 1,2,4-triazolo[4,3-*a*]pyrimidin-5-one derivatives [33]. Additionally, treatment of 5-(3-(furan-2-yl)-1-phenyl-1*H*-pyrazol-4-yl)-1,3-dimethyl-7-thioxo-5,6,7,8-tetrahydropyrimido[4,5-*d*]pyrimidine-2,4(1*H*,3*H*)-dione (**27**) with the hydrazonoyl chlorides **5a** and **5d** in boiling chloroform in the presence of triethylamine afforded ethyl 5-(3-(furan-2-yl)-1-phenyl-1*H*-pyrazol-4-yl)-1,3-dimethyl-2,4-dioxo-7-phenyl-1,2,3,4,5,7-hexahydro-pyrimido[5,4-*e*][1,2,4]triazolo[4,3-*a*]pyrimidine-9-carboxylate (**28a**) and 5-(3-(furan-2-yl)-1-phenyl-1*H*-pyrazol-4-yl)-1,3-dimethyl-2,4-dioxo-N,7-diphenyl-1,2,3,4,5,7-hexahydro-pyrimido[5,4-*e*][1,2,4]-triazol[4,3-*a*]pyrimidine-9-carboxamide (**28b**), respectively (Scheme 8).

## 3. Antimicrobial Activity

Twenty seven of the newly synthesized target compounds were evaluated for their in vitro antibacterial activity against *Staphylococcus* *aureus* and *Bacillus subtilis* as examples of Gram-positive bacteria and *Pseudomonas aeruginosa* and *Escherichia coli* as examples of Gram-negative bacteria. They were also evaluated for their in vitro antifungal activity against a representative panel of fungal strains i.e., *Aspergillus fumigatus*, and *Candida albicans*. Ampicillin, Gentamicin and Amphotericin B are used as reference drugs for in vitro antibacterial activity and for in vitro antifungal activity, respectively, at The Regional Center for Mycology and Biotechnology at Al-Azhar University (Nasr City, Cairo, Egypt). The results of testing for antimicrobial effects are summarized in Table 1, Table 2 and Table 3.

Compound **9a** had no activity against *Aspergillus fumigates*, *Candida albicans*, *Streptococcus pneumonia*, *Bacillus subtilis*, *Pseudomonas aeruginosa*, and *Escherichia coli*.

- *Candida albicans* and *Pseudomonas aeruginosa* were resistant to compounds **9a**, **9b**, **9c**, **9d**, **10a**, **10b**, **10c**, **10d**, and **14f**.
- *Aspergillus fumigatus* was susceptible to compounds **13b**, **14b**, **15b**, and **15c** when compared to the Amphotericin B standard.
- *Candida albicans* was susceptible to compound **13b** when compared to the Amphotericin B standard.
- *Streptococcus pneumoniae* was susceptible to compounds **13b**, and **14b** when compared to the Ampicillin standard.
- *Bacillus subtilis* was susceptible to compounds **13b**, **14b**, and **15b** when compared to the Ampicillin standard.
- *Pseudomonas aeruginosa* was susceptible to compounds **13b**, **15a**, and **26d** when compared to their standard Gentamicin.
- *Escherichia coli* was susceptible to compounds **10a**, **13b**, **14b**, **15f**, and **28a** when compared to the Gentamicin standard.

The minimum inhibitory concentration (MIC) is the lowest concentration of an antimicrobial that will inhibit the visible growth of a microorganism after overnight incubation. Minimum inhibitory concentrations are important in diagnostic laboratories to confirm resistance of microorganisms to an antimicrobial agent and also to determine the potency of new antimicrobial agents [34]. A MIC is generally regarded as the most basic laboratory measurement of the activity of an antimicrobial agent against an organism [35]. MIC was determined by the broth micro dilution method using 96-well micro-plates [36,37]. *Pseudomonas aeruginosa* showed an the same as the MIC value of 12.5 mg/mL of the tested compound **13b** suggesting high inhibitory activity compared to that of Gentamicin. Also, compound **10a** showed an MIC value of 3.9 against *Escherichia coli* which was the same MIC value of Gentamicin against *Escherichia coli* (Table 2).

The half maximal inhibitory concentration (IC_50_) is a measure of the effectiveness of a substance in inhibiting a specific biological or biochemical function. This quantitative measure indicates how much of a particular substance is needed to inhibit a given biological process by half. And here the biological function is the growth of microorganisms *Aspergillus fumigatus*, *Syncephalastrum racemosum*, *Geotrichum candidum, Candida albicans*, *Streptococcus pneumoniae*, *Bacillus subtilis*, *Pseudomonas aeruginosa*, and *Escherichia coli* (Table 3).

## 4. Experimental Section

### 4.1. General Information

All melting points were determined on an Electrothermal apparatus (Bibby Sci. Lim. Stone, Staffordshire, UK) and are uncorrected. IR spectra were recorded (KBr discs) on a FT-IR 8201 PC spectrophotometer (Shimadzu, Tokyo, Japan). ^1^H-NMR spectra were recorded in CDCl_3_ and DMSO-*d*_6_ solutions on a Gemini 300 MHz spectrometer (Varian, Mercury VX-300 NMR spectrometer, Bruker BioSpin GmbH, Rheinstetten, Germany) and chemical shifts are expressed in δ ppm units using TMS as an internal reference. Mass spectra were recorded on a Shimadzu GC-MS QP1000 EX instrument. (Tokyo, Japan) Elemental analyses were carried out at the Microanalytical Center of Cairo university. Hydrazonoyl halides **5a**–**d** were prepared as previously reported [38,39,40,41]. Antimicrobial screening was performed at the Regional Center for Mycology and Biotechnology, Al-Azhar University, Cairo, Egypt.

#### 4.1.1. General Procedure for the Synthesis of Thiadiazoles **9a**–**c** and **10a**–**c**

Mixtures of alkyl carbodithioate (**3a**, **3b**, **4a** or **4b**) (5 mmol), hydrazonoyl halides (**5a**, **5b**, or **5c**) (5 mmol) and triethylamine (0.75 mL, 5 mmol) in ethanol (20 mL) were stirred at room temperature for 3 h. The resulting solid was collected and recrystallized from acetic acid (dioxane) to give **9a**–**c** and **10a**–**c**, respectively, in good yields.

*Ethyl (Z)-5-(((Z)-(1,1’-diphenyl-3'-(p-tolyl)-1H,1'H-[3,4’-bipyrazol]-4-yl)methylene)hydrazono)-4-phenyl-4,5-dihydro-1,3,4-thiadiazole-2-carboxylate* (**9a**). Yellow solid from glacial acetic acid, yield (1.9 g, 75%), mp: 176–177 °C; IR (KBr, cm^−1^): 3078 (=C–H), 2993–2862 (–C–H), 1708 (–C=O), 1608 (–C=N); ^1^H-NMR: δ 1.45 (t, 3H, –CH_2_CH_3_), 2.43 (s, 3H, 4-CH_3_C_6_H_4_), 4.44 (q, 2H, –OCH_2_CH_3_), 7.26–7.97 (m, 18H, Ar–H), 8.95 (s, 1H pyrazole-H-5); MS (*m*/*z*): 510 (M + 2, 9), 509 (M + 1, 32), 508 (M^+^, 72), 259 (52), 246 (41), 91 (57), 77 (100); Anal. Calcd. for C_28_H_24_N_6_O_2_S (508.59): C, 66.12; H, 4.76; N, 16.52; S, 6.30; found: C, 66.10; H, 4.75; N, 16.53; S, 6.33.

*1-((Z)-5-(((Z)-(1,1'-Diphenyl-3'-(p-tolyl)-1H,1'H-[3,4'-bipyrazol]-4-yl)methylene)hydrazono)-4-phenyl-4,5-dihydro-1,3,4-thiadiazol-2-yl)ethan-1-one* (**9b**). Yellow solid from glacial acetic acid, yield (2 g, 84%), mp: 165–166 °C; IR (KBr, cm^−1^): 3040 (=C–H), 2869 (–C–H), 1674 (–C=O), 1604 (–C=N); ^1^H-NMR: δ 2.43 (s, 3H, 4-CH_3_C_6_H_4_), 2.68 (s, 3H, –CO–CH_3_), 7.27–8.02 (m, 14H, Ar–H), 8.50 (s, 1H, –C=H), 8.85 (s, 1H, pyrazole-H-5); MS (*m*/*z*): 479 (M + 1, 25), 478 (M^+^, 32), 310 (35), 307 (34), 150 (33), 138 (38), 126 (30), 122 (39), 91 (37), 77 (26); *Anal.* Calcd. for C_27_H_22_N_6_OS (478.57): C, 67.75; H, 4.61; N, 17.58; S, 6.70; found: C, 67.76; H, 4.63; N, 17.56; S, 6.71.

*((Z)-5-(((Z)-(1,1'-Diphenyl-3'-(p-tolyl)-1H,1'H-[3,4’-bipyrazol]-4-yl)methylene)hydrazono)-4-phenyl-4,5-dihydro-1,3,4-thiadiazol-2-yl)(phenyl)methanone* (**9c**). Orange solid from dioxane, yield (2.27 g, 84%), mp: 264–265 °C; IR (KBr, cm^−1^): 2863 (C–H), 1700 (–C=O), 1608 (–C=N); ^1^H-NMR: δ 2.44 (s, 3H, 4-CH_3_C_6_H_4_), 7.27–8.49 (m, 19H, Ar–H), 8.54 (s, 1H, –C=H), 8.96 (s, 1H, pyrazole-H-5); MS (*m*/*z*): 542 (M + 2, 57), 541 (M + 1, 59), 540 (M^+^, 68), 525 (55), 510 (53), 494 (46), 479 (54), 468 (70), 458 (51), 439 (54), 421 (60), 412 (62), 371 (100), 355 (53), 282 (78), 135 (64), 105 (75); Anal. Calcd. for C_32_H_24_N_6_OS (540.64): C, 71.09; H, 4.47; N, 15.54; S, 5.93; found: C, 71.07; H, 4.48; N, 15.54; S, 5.95.

*Ethyl (Z)-5-(((Z)-(3'-(furan-2-yl)-1,1'-diphenyl-1H,1'H-[3,4'-bipyrazol]-4-yl)methylene)hydrazono)-4-phenyl-4,5-dihydro-1,3,4-thiadiazole-2-carboxylate* (**10a**). Yellow solid from ethanol, yield (2.2 g, 91%), mp: 171–173 °C; IR (KBr, cm^−1^): 3108 (=C-H), 2980 (–C–H), 1738 (–C=O), 1603 (–C=N), 1545 (C=C); ^1^H-NMR: δ 1.44 (t, 3H, –CH_2_CH_3_), 4.45 (q, 2H, –OCH_2_CH_3_), 6.55 (d, 1H, furyl-H), 6.94 (q, 1H, furyl-H), 7.27–8.03 (m, 11H, Ar–H + 1furyl-H), 8.47 (s, 1H, CH=N), 8.89 (s, 1H, pyrazole-H-5); MS (*m*/*z*): 485 (M + 1, 9), 484 (M^+^, 22), 221 (100), 135 (30), 120 (27), 92 (46), 78 (60); Anal. Calcd. for C_25_H_20_N_6_O_3_S (484.53): C, 61.97; H, 4.16; N, 17.34; S, 6.62; found: C, 61.99; H, 4.15; N, 17.35; S, 6.60.

*1-((Z)-5-(((Z)-(3'-(Furan-2-yl)-1,1'-diphenyl-1H,1'H-[3,4'-bipyrazol]-4-yl)methylene)hydrazono)-4-phenyl-4,5-dihydro-1,3,4-thiadiazol-2-yl)ethan-1-one* (**10b**). Yellow solid from glacial acetic acid, yield (1.3 g, 65%), mp: 200–202 °C; IR (KBr, cm^−1^): 3021 (=C–H), 2918 (–C–H), 1683 (C=O), 1604 (C=N), 1548 (C=C); ^1^H-NMR: δ 2.63 (s, 3H, CO–CH_3_), 6.55 (q, 1H, furyl), 6.89 (d, 1H, furyl), 7.27–7.96 (m, 11H, Ar–H + 1furyl-H), 8.28 (s, 1H, N=CH), 8.91 (s, 1H, pyrazole-H-5); MS (*m*/*z*): 454 (M^+^, 33), 426 (100), 221 (34), 193 (16), 119 (14), 92 (12), 78 (23), 65 (15); Anal. Calcd. for C_24_H_18_N_6_O_2_S (404.50): C, 63.40; H, 3.98; N, 18.50; S, 7.05; found: C, 63.42; H, 3.99; N, 18.49; S, 7.07.

*5-(-((3-(Furan-2-yl)-1-phenyl-1H-pyrazol-4-yl)methylene)hydrazono)-4-phenyl-4,5-dihydro-1,3,4-thiadiazol-2-yl)(phenyl)methanone* (**10c**). Red solid from glacial acetic acid, yield (2.1 g, 83%), mp: 228–230 °C; IR (KBr, cm^−1^): 3061 (=C–H), 1725 (–C=O), 1601 (–C=N), 1547 (C=C); ^1^H-NMR: δ 6.56 (q, 1H, furyl-H), 6.85 (d, 1H, furyl-H), 7.27–8.35 (m, 16H, Ar–H + 1furyl-H), 8.35 (s, 1H, CH=N), 8.90 (s, 1H, pyrazole-H-5); MS (*m*/*z*): 518 (M + 2, 1), 517 (M + 1, 5), 516 (M^+^, 31), 502 (12), 487 (97), 167 (10), 135 (25), 131 (10), 235 (17), 221 (49), 193 (24), 129 (17), 119 (11), 106 (100), 78 (45), 65 (30), 51 (45); Anal. Calcd. for C_29_H_20_N_6_O_2_S (516.57): C, 67.43; H, 3.90; N, 16.27; S, 6.21; found: C, 67.40; H, 3.89; N, 16.27; S, 6.23.

#### 4.1.2. General Procedure for the Synthesis of Chalcones **12a**–**f**

10% NaOH solution (0.2 g, 10 mL, 5 mmol) was added dropwise to a mixture of the appropriate 2-acetylfuran (0.54 g, 5 mmol) or 4-methylacetophenone (0.67 mL, 5 mmol) and the appropriate of 1-phenyl-3-(*p*-tolyl)-1*H*-pyrazole-4-carbaldehyde (**1a**) (1.3 g, 5 mmol), 3-(furan-2-yl)-1-phenyl-1*H*-pyrazole-4-carbaldehyde (1.2 g, 5 mmol) (**1b**) or furfuraldehyde (**1c**) (0.48 g, 5 mmol) in ethanol (30 mL), at 0–5 °C while stirring. The precipitate that formed was filtered, washed with ethanol (10 mL), and recrystallized from ethanol to give **12a**–**f**, respectively.

*(E)-3-(Furan-2-yl)-1-(p-tolyl)prop-2-en-1-one* (**12a**). Mp: 64–65 °C (lit. mp: 62–64 °C) [42].

*(E)-1-(Furan-2-yl)-3-(3-(furan-2-yl)-1-phenyl-1H-pyrazol-4-yl)prop-2-en-1-one* (**12b**). Yellow solid from ethanol, yield (1.5 g, 90%), mp: 155–156 °C; IR (KBr, cm^−1^): 3103 (=C–H), 1652 (C=O); ^1^H-NMR: δ 6.54 (q, 1H, furyl-H), 6.59 (q, 1H, furyl-H), 6.90 (q, 1H, furyl-H), 7.27–8.33 (m, 11H, ArH’s + 2CH=CH + 3furyl-H + pyrazole-H-5); MS (*m*/*z*): 332 (M + 2, 2), 331 (M + 1, 16), 330 (M^+^, 100), 314 (1), 300 (22), 242 (4), 270 (25), 214 (5), 91 (7); Anal. Calcd. for C_20_H_14_N_2_O_3_ (330.34): C, 72.72; H, 4.27; N, 8.48; found: C, 72.69; H, 4.28; N, 8.49.

*(E)-3-(3-(Furan-2-yl)-1-phenyl-1H-pyrazol-4-yl)-1-(p-tolyl)prop-2-en-1-one* (**12c**). Yellow solid, yield (1.63 g, 92%), mp: 146–147 °C; IR (KBr, cm^−1^): 3122 (=C-H aromatic), 3069 (=C–H), 2919 (–C–H), 1651 (C=O); ^1^H-NMR: δ 2.44 (s, 3H, 4-CH_3_C_6_H_4_), 6.54 (q, 1H, furyl-H), 6.89 (d, 1H, furyl-H), 7.26–8.19 (m, 12H, Ar–H’s + 2H + 1furyl-H), 8.25 (s, 1H, pyrazole-H-5); MS (*m*/*z*): 355 (M + 1, 2), 354 (M^+^, 7), 308 (20), 281 (25), 234 (43), 209 (27), 208 (42), 207 (28), 181 (13), 180 (43), 179 (20), 178 (15), 168 (16), 167 (36), 166 (100), 154 (13), 153 (44), 152 (32), 140 (38), 139 (10), 127 (13), 126 (10), 115 (16), 114 (10), 113 (13), 63 (10), 29 (30), 27 (13); Anal. Calcd. for C_23_H_18_N_2_O_2_ (354.40): C, 77.95; H, 5.12; N, 7.90; found: C, 77.97; H, 5.10; N, 7.91.

*(E)-3-(1-Phenyl-3-(p-tolyl)-1H-pyrazol-4-yl)-1-(p-tolyl)prop-2-en-1-one* (**12d**). mp: 135–136 °C (lit. mp.: 101–103 °C) [43].

*(E)-1,3-di(Furan-2-yl)prop-2-en-1-one* (**12e**). Mp: 89–90 °C (lit. mp: 88–90 °C) [39].

*1-(Furan-2-yl)-3-(1-phenyl-3-(p-tolyl)-1H-pyrazol-4-yl)prop-2-en-1-one* (**12f**) Yellow solid, yield (1.5 g, 86%), mp: 154–155 °C; IR (KBr, cm^−1^): 3138 (=C–H aromatic), 3105 (=C–H), 2922 (C–H), 1649 (C=O); ^1^H-NMR: δ 2.44 (s, 3H, 4-CH_3_C_6_H_4_), 6.56 (q, 1H, furyl-H), 7.23–7.97 (m, 13H, ArH’s + 2CH=CH + 2furyl-H), 8.35 (s, 1H, pyrazole-H-5); MS (*m*/*z*): 356 (M + 2, 3), 355 (M + 1, 20), 354 (100), 339 (11), 273 (17), 188 (10), 186 (20), 172 (20), 171 (24), 170 (17), 157 (10), 156 (17), 143 (11), 142 (11), 130 (24), 129 (14), 128 (15); Anal. Calcd. for C_23_H_18_N_2_O_2_ (354.40): C, 77.95; H, 5.12; N, 7.90; found: C, 77.97; H, 5.14; N, 7.88.

#### 4.1.3. Synthesis of Carbothioamide Derivatives **13a**–**f**

Mixtures of chalcones **12a**–**f** (5 mmol) and thiosemicarbazide (0.46 g, 5 mmol) in ethanol (20 mL) were refluxed for 3 h. The resulting solid was collected and recrystallized from acetic acid to give **13a**–**f**, respectively.

*5-(Furan-2-yl)-3-(p-tolyl)-4,5-dihydro-1H-pyrazole-1-carbothioamide* (**13a**). Mp: 190–191 °C. (lit. mp: 280–282 °C) [44].

*3’,5-di(Furan-2-yl)-1'-phenyl-3,4-dihydro-1'H,2H-[3,4'-bipyrazole]-2-carbothioamide* (**13b**). Yellow solid, yield (1.7 g, 85%), mp: 285–287 °C; IR (KBr, cm^−1^): 3334 (N–H), 3120 (=C–H); ^1^H-NMR: δ 3.15 (dd, 1H, pyrazoline-H), 3.90 (q, 1H, pyrazoline-H), 6.20 (dd, 1H, pyrazoline-H), 6.65 (m, 2H, furyl-H), 6.67 (q, 1H, furyl-H), 7.05 (d, 1H, furyl-H), 7.44–7.91 (m, 9H, Ar–H + 1furyl-H + 2N–H), 8.95 (s, 1H, pyrazole-H-5); MS (*m*/*z*): 404 (M + 1, 2), 403 (M^+^, 9), 300 (6), 256 (1), 228 (10), 209 (36), 196 (100), 181 (58), 164 (50), 136 (25), 121 (6), 93 (9), 77 (7); Anal. Calcd. for C_21_H_17_N_5_O_2_S (403.46): C, 62.52; H, 4.25; N, 17.36; S, 7.95; found: C, 62.55; H, 4.26; N, 17.34; S, 7.94.

*3’-(Furan-2-yl)-1'-phenyl-5-(p-tolyl)-3,4-dihydro-1'H,2H-[3,4'-bipyrazole]-2-carbothioamide* (**13c**). Yellow solid, yield (1.7 g, 80%), mp: 263–265 °C; IR (KBr, cm^−1^): 3265 (N–H), 3136 (=C–H aromatic), 3048 (=C–H), 2917 (–C–H); ^1^H-NMR: δ 2.34 (s, 3H, 4-CH_3_C_6_H_4_), 3.18 (dd, 1H, pyrazoline-H), 3.93 (q, 1H, pyrazoline-H), 6.16 (dd, 1H, pyrazoline-H), 6.65–7.84 (m, 14H, Ar–H + 3furyl-H + 2N–H), 8.08 (s, 1H, pyrazole-H-5); MS (*m*/*z*): 427 (M^+^, 5), 408 (11), 386 (18), 344 (25), 302 (48), 260 (100), 231 (6), 203 (3), 153 (1); Anal. Calcd. for C_24_H_21_N_5_OS (427.52): C, 67.43; H, 4.95; N, 16.38; S, 7.50; found: C, 67.39; H, 4.95; N, 16.38; S, 7.52.

*1’-Phenyl-3’,5-di-p-tolyl-3,4-dihydro-1'H,2H-[3,4'-bipyrazole]-2-carbothioamide* (**13d**). White solid from dioxane, yield (1.7 g, 75%), mp: 276–277 °C; IR (KBr, cm^−1^): 3430; 3115 (N–H), 3046 (=C–H aromatic), 2918 (–C–H); ^1^H-NMR: δ 2.34 (s, 3H, 4-CH_3_C_6_H_4_), 2.35 (s, 3H, 4-CH_3_C_6_H_4_), 3.18 (dd, 1H, pyrazoline-H), 3.93 (q, 1H, pyrazoline-H), 6.16 (dd, 1H, pyrazoline-H), 6.66–7.82 (m, 15H, Ar–H + 2N–H), 8.07 (s, 1H, pyrazole-H-5); MS (*m*/*z*): 452 (M + 1, 6), 253 (17), 250 (13), 225 (16), 221 (10), 206 (19), 193 (20), 174 (14), 151 (9), 142 (29), 116 (10), 110 (16), 108 (17), 107 (16), 91 (100), 84 (43), 82 (26), 79 (20), 82 (26), 77 (12); Anal. Calcd. for C_27_H_25_N_5_S (451.59): C, 71.81; H, 5.58; N, 15.51; S, 7.10; found: C, 71.79; H, 5.57; N, 15.52; S, 7.12.

*3,5-di(Furan-2-yl)-4,5-dihydro-1H-pyrazole-1-carbothioamide* (**13e**). Mp: 164–166 °C. (lit. mp: 162–163 °C) [45].

*5-(Furan-2-yl)-1'-phenyl-3'-(p-tolyl)-3,4-dihydro-1'H,2H-[3,4'-bipyrazole]-2-carbothioamide* (**13f**). White solid, yield (1.6 g, 76%), mp: 247–248 °C;IR (KBr, cm^−1^): 3255 (N–H), 3145 (=C–H), 2919 (–C–H); ^1^H-NMR: δ 2.37 (s, 3H, 4-CH_3_C_6_H_4_), 3.05 (dd, 1H, pyrazoline-H), 3.85 (q, 1H, pyrazoline-H), 6.06 (dd, 1H, pyrazoline-H), 6.65–7.90 (m, 14H, Ar–H + 3furyl-H + 2N–H), 8.01 (s, 1H, pyrazole-H-5); MS (*m*/*z*): 427 (M+, 7), 384 (8), 354 (10), 325 (9), 296 (6), 270 (5), 254 (11), 240 (8), 213 (9), 103 (100), 75 (32); Anal. Calcd. for C_24_H_21_N_5_OS (427.52): C, 67.43; H, 4.95; N, 16.38; S, 7.50; found: C, 67.41; H, 4.95; N, 16.37; S, 7.51.

#### 4.1.4. 5-Arylazothiazole Derivatives **14a**–**f** and **15a**–**f**

##### Method A 

Mixtures of the appropriate thioamides **13a**–**f** (5 mmol), the appropriate **5b** and **5c** (5 mmol) and triethylamine (0.75 mL, 5 mmol) in ethanol (20 mL) were refluxed for 3 h. The resulting solid was collected and recrystallized from acetic acid (or dioxane) to give **14a**–**f** and **15a**–**f**, respectively.

##### Method B

Benzenediazonium chloride (5 mmol), which was prepared from aniline (0.45 mL, 5 mmol), hydrochloric acid (6 N, 6 mL), and sodium nitrite (0.35 g, 5 mmol), was added dropwise with stirring to a cold solution of 2-(5-(furan-2-yl)-3-(*p*-tolyl)-4,5-dihydro-1*H*-pyrazol-1-yl)-4-phenylthiazole (**16**) (1.92 g, 5 mmol) and sodium acetate trihydrate (1.3 g, 10 mmol) in ethanol (50 mL). The reaction mixture was stirred in an ice bath at 0–5 °C for 3 h. The result solid was collected and recrystallized to give a product identical in all aspects (mp, mixed mp. and spectra) with **15a**.

*(E)-2-(3-(Furan-2-yl)-5-(p-tolyl)-4,5-dihydro-1H-pyrazol-1-yl)-4-methyl-5-(phenyldiazenyl)-thiazole* (**14a**). Red solid, yield (1.5 g, 70%), mp: 179–180 °C; IR (KBr, cm^−1^): 2920 (–C–H); ^1^H-NMR: δ 2.37 (s, 3H, 4-CH_3_C_6_H_4_), 2.50 (s, 3H, –CH_3_), 3.56 (dd, 1H, pyrazoline-H), 3.90 (dd, 1H, pyrazoline-H), 5.90 (dd, 1H, pyrazoline-H), 6.43 (dd, 1H, furyl-H), 6.52 (dd, 1H, furyl-H), 7.31–7.76 (m, 10H, Ar–H + 1furyl-H); MS (*m*/*z*): 427 (M+, 1), 326 (12), 236 (16), 232 (14), 206 (39), 204 (11), 194 (31), 147 (51), 146 (14), 134 (23), 133 (41), 129 (11), 121 (32), 118 (12), 117 (100), 115 (10), 107 (24), 91 (17), 73 (24); Anal. Calcd. for C_24_H_21_N_5_OS (427.52): C, 67.43; H, 4.95; N, 16.38; S, 7.50; found: C, 67.41; H, 4.94; N, 16.37; S, 7.51.

*(E)-2-(3',5-Di(furan-2-yl)-1'-phenyl-3,4-dihydro-1'H,2H-[3,4'-bipyrazol]-2-yl)-4-methyl-5-(phenyldiazen-yl)-thiazole* (**14b**). Red solid, yield (1.5 g, 55%), mp: 275–277 °C; IR (KBr, cm^−1^): 3146 (=C–H), 2922 (–C–H); ^1^H-NMR: δ 2.49 (s, 3H, –CH_3_), 3.13 (dd, 1H, pyrazoline-H), 3.97 (dd, 1H, pyrazoline-H), 6.17 (dd, 1H, pyrazoline-H), 6.65–7.91 (m, 16H, Ar–H + 3-furyl-H), 8.95 (s, 1H, pyrazole-H-5); MS (*m*/*z*): 545 (4), 520 (14), 505 (35), 504 (10), 492 (13), 491 (17), 478 (10), 460 (10), 270 (48), 252 (17), 241 (18), 223 (10), 176 (19), 121 (40), 106 (11), 83 (10), 71 (11); Anal. Calcd. for C_30_H_23_N_7_O_2_S (545.61): C, 66.04; H, 4.25; N, 17.97; S, 5.88; found: C, 66.02; H, 4.24; N, 17.98; S, 5.89.

*(E)-2-(3’-(Furan-2-yl)-1’-phenyl-5-(p-tolyl)-3,4-dihydro-1'H,2H-[3,4’-bipyrazol]-2-yl)-4-methyl-5-(phenyl-diazenyl)-thiazole* (**14c**). Red solid, yield (1.7 g, 60%), mp: 250–252 °C; IR (KBr, cm^−1^): 3139 (=C–H), 2917 (–C–H); ^1^H-NMR: δ 2.30 (s, 3H, 4-CH_3_C_6_H_4_), 2.50 (s, 3H, –CH_3_), 3.18 (dd, 1H, pyrazoline-H), 3.97 (dd, 1H, pyrazoline-H), 6.19 (dd, 1H, pyrazoline-H), 6.65–7.88 (m, 17H, Ar–H + 3furyl-H), 8.08 (s, 1H, pyrazole-H-5); MS (*m*/*z*): 569 (M+, 4), 484 (16), 454 (14), 358 (42), 317 (60), 289 (34), 230 (34), 130 (16), 103 (26), 77 (14); Anal. Calcd. for C_33_H_27_N_7_OS (569.68): C, 69.57; H, 4.78; N, 17.21; S, 5.63; found: C, 69.55; H, 4.78; N, 17.20; S, 5.64.

*(E)-4-Methyl-2-(1'-phenyl-3',5-di-p-tolyl-3,4-dihydro-1'H,2H-[3,4'-bipyrazol]-2-yl)-5-(phenyldiazenyl)-thiazole* (**14d**). Orange solid, yield (2.1 g, 70%), mp: 210–211 °C; IR (KBr, cm^−1^): 3136 (=C–H aromatic), 3051 (=C–H), 2950 (–C–H); ^1^H-NMR: δ 2.40 (s, 6H, 4-CH_3_C_6_H_4_), 2.63 (s, 3H, –CH_3_), 3.65; 3.9 (m, 2H, pyrazoline-H), 5.1 (q, 1H, pyrazoline-H), 6.99–8.95 (m, 19H, Ar–H + pyrazole-H-5); MS (*m*/*z*): 593 (M+, 2), 581 (12), 578 (16), 574 (35), 300 (33), 299 (100), 298 (11), 288 (11), 287 (19), 286 (77), 285 (15), 241 (10), 227 (24), 211 (18); Anal. Calcd. for C_36_H_31_N_7_S (593.74): C, 72.82; H, 5.26; N, 16.51; S, 5.40; found: C, 72.85; H, 5.27; N, 16.49; S, 5.39.

*(E)-2-(3,5-Di(furan-2-yl)-4,5-dihydro-1H-pyrazol-1-yl)-4-methyl-5-(phenyldiazenyl)-thiazole* (**14e**). Red solid, yield (1.3 g, 66%), mp: 201–203 °C; IR (KBr, cm^−1^): 3122 (=C–H), 2950 (–C–H); ^1^H-NMR: δ 2.54 (s, 3H, –CH_3_), 3.50 (dd, 1H, pyrazoline-H), 3.89 (dd, 1H, pyrazoline-H), 5.90 (dd, 1H, pyrazoline-H), 6.43–6.73 (m, 3H, furyl-H), 7.14 (t, 1H, furyl-H), 7.37–7.97 (m, 7H, Ar–H +2 furyl-H); MS (*m*/*z*): 405 (M+2, 3), 404 (M + 1, 21), 403 (M+, 75), 388 (41), 361 (64), 275 (12), 146 (7), 130 (14); Anal. Calcd. for C_21_H_17_N_5_O_2_S (403.46): C, 62.52; H, 4.25; N, 17.36; S, 7.95; found: C, 62.49; H, 4.25; N, 17.37; S, 7.96.

*(E)-2-(5-(Furan-2-yl)-1'-phenyl-3'-(p-tolyl)-3,4-dihydro-1'H,2H-[3,4’-bipyrazol]-2-yl)-4-methyl-5-(phenyldiazenyl)-thiazole* (**14f**). Orange solid, yield (2.1 g, 75%), mp: 216–219 °C; IR (KBr, cm^−1^): 3120 (=C–H), 2914 (–C–H); ^1^H-NMR: δ 2.39 (s, 3H, 4-CH_3_C_6_H_4_), 2.47 (s, 3H, –CH_3_), 4.49 (q, 2H, pyrazoline-H), 5.95 (q, 1H, pyrazoline-H), 6.70 (dd, 1H, furyl-H), 7.05 (d, 1H, furyl-H),7.28–7.61 (m, 15H, Ar–H + 1furyl-H), 8.52 (s, 1H, pyrazole-H-5); MS (*m*/*z*): 569 (M+, 7), 553 (10), 398 (12), 370 (15), 248 (51), 235 (100), 91 (27); Anal. Calcd. for C_33_H_27_N_7_OS (569.68): C, 69.57; H, 4.78; N, 17.21; S, 5.63; found: C, 69.58; H, 4.79; N, 17.22; S, 5.62.

*(E)-2-(5-(Furan-2-yl)-3-(p-tolyl)-4,5-dihydro-1H-pyrazol-1-yl)-4-phenyl-5-(phenyldiazenyl)-thiazole* (**15a**). Orange solid, yield (1.9 g, 76%), mp: 233–234 °C; IR (KBr, cm^−1^): 3147 (=C–H), 2930 (–C–H); ^1^H-NMR: δ 2.38 (s, 3H, 4-CH_3_C_6_H_4_), 3.60 (dd, 1H, pyrazoline-H), 4.00 (dd, 1H, pyrazoline-H), 6.00 (dd, 1H, pyrazoline-H), 6.46 (q, 1H, furyl-H), 6.65 (d, 1H, furyl-H), 7.33–8.05 (m, 15H, Ar–H + 1furyl-H); MS (*m*/*z*): 489 (M+, 2), 428 (10), 335 (18), 321 (10), 293 (18), 163 (11), 165 (9), 155 (100), 92 (10), 91 (68), 43 (9); Anal. Calcd. for C_29_H_23_N_5_OS (489.59): C, 71.14; H, 4.74; N, 14.30; S, 6.55; found: C, 71.15; H, 4.73; N, 14.31; S, 6.52.

*(E)-2-(3',5-di(Furan-2-yl)-1'-phenyl-3,4-dihydro-1'H,2H-[3,4'-bipyrazol]-2-yl)-4-phenyl-5-(phenyldiazenyl)-thiazole* (**15b**). Orange solid, yield (1.8 g, 60%), mp: 270–272 °C; IR (KBr, cm^−1^): 3150 (=C–H); ^1^H-NMR: δ 3.08 (q, 1H, pyrazoline-H), 3.96 (q, 1H, furyl-H), 6.18 (q, 1H, pyrazoline-H), 6.66–8.10 (m, 22H, Ar–H + pyrazole-H-5 + 6furyl-H); MS (*m*/*z*): 607 (M+, 2), 331 (14), 284 (100), 169 (94), 127 (17), 109 (57), 43 (86); Anal. Calcd. for C_35_H_25_N_7_O_2_S (607.68): C, 69.18; H, 4.15; N, 16.13; S, 5.28; found: C, 69.19; H, 4.16; N, 16.15; S, 5.23.

*(E)-2-(3'-(Furan-2-yl)-1'-phenyl-5-(p-tolyl)-3,4-dihydro-1'H,2H-[3,4'-bipyrazol]-2-yl)-4-phenyl-5-(phenyldiazenyl)-thiazole* (**15c**). Orange solid, yield (2.3 g, 74%), mp: 240–242 °C; IR (KBr, cm^−1^): 3139 (=C–H), 2920 (–C–H); ^1^H-NMR: δ 2.34 (s, 3H, 4-CH_3_C_6_H_4_), 3.18 (dd, 1H, pyrazoline-H), 3.39 (dd, 1H, pyrazoline-H), 6.11 (dd, 1H, pyrazoline-H), 6.64–8.08 (m, 23H, Ar–H + pyrazole-H-5 + 3furyl-H); MS (*m*/*z*): 631 (M+, 4), 477 (9), 409 (65), 297 (6), 271 (5), 245 (8), 227 (11), 203 (13), 149 (17), 135 (49), 107 (31), 69 (100); Anal. Calcd. for C_38_H_29_N_7_OS (631.75): C, 72.25; H, 4.63; N, 15.52; S, 5.08; found: C, 72.25; H, 4.62; N, 15.50; S, 5.10.

*(E)-4-Phenyl-2-(1'-phenyl-3',5-di-p-tolyl-3,4-dihydro-1'H,2H-[3,4'-bipyrazol]-2-yl)-5-(phenyldiazenyl)-thiazole* (**15d**). Red solid, yield (1.5 g, 45%), mp: 208–209 °C; IR (KBr, cm^−1^): 3142 (=C–H), 2924 (–C–H); ^1^H-NMR: δ 2.50 (s, 6H, 4-CH_3_C_6_H_4_), 3.36 (q, 1H, pyrazoline-H), 3.63 (q, 1H, pyrazoline-H), 5.30 (q, 1H, pyrazoline-H), 7.36–9.00 (m, 24H, Ar–H + pyrazole-H-5); MS (*m*/*z*): 655 (M+, 1), 498 (10), 339 (10), 281 (17), 243 (51), 242 (62), 210 (12), 171 (32), 156 (25), 73 (35), 71 (76), 41 (26), 39 (14), 27 (19); *Anal.* Calcd. for C_41_H_33_N_7_S (655.81): C, 75.09; H, 5.07; N, 14.95; S, 4.89; found: C, 75.10; H, 5.05; N, 14.96; S, 4.88.

*(E)-2-(3,5-di(Furan-2-yl)-4,5-dihydro-1H-pyrazol-1-yl)-4-phenyl-5-(phenyldiazenyl)-thiazole* (**15e**). Red solid, yield (1.3 g, 57%), mp: 181–183 °C; IR (KBr, cm^−1^): 3100 (=C–H); ^1^H-NMR: δ 4.00 (m, 2H, pyrazoline-H), 6.00 (q, 1H, pyrazoline-H), 6.46; 6.64; 6.74 (m, 3H, furyl-H), 7.16 (d, 1H, furyl-H), 7.41–8.27 (m, 12H, Ar–H + 2furyl-H); MS (*m*/*z*): 465 (M+, 3), 271 (29), 253 (70), 233 (16), 221 (100), 105 (10); Anal. Calcd. for C_26_H_19_N_5_O_2_S (465.53): C, 67.08; H, 4.11; N, 15.04; S, 6.89; found: C, 67.10; H, 4.09; N, 15.05; S, 6.86.

*(E)-2-(5-(Furan-2-yl)-1'-phenyl-3'-(p-tolyl)-3,4-dihydro-1'H,2H-[3,4'-bipyrazol]-2-yl)-4-phenyl-5-(phenyldiazenyl)-thiazole* (**15f**). Orange, yield (1.7 g, 53%), mp: 249–250 °C; IR (KBr, cm^−1^): 3150 (=C–H), 2922 (–C–H); ^1^H-NMR: δ 2.37 (s, 3H, 4-CH_3_C_6_H_4_), 3.30 (dd, 1H, pyrazoline-H), 3. 90 (dd, 1H, pyrazoline-H), 6.16 (q, 1H, pyrazoline-H), 6.65 (q, 1H, furyl-H), 7.01 (q, 1H, furyl-H), 7.82–8.10 (m, 21H, Ar–H + pyrazole-H-5 + 1furyl-H); MS (*m/z*): 633 (M + 2, 1), 632 (M + 1, 8), 631 (M+, 28), 630 (60), 380 (7), 337 (14), 322 (37), 321 (100), 310 (47), 292 (13), 109 (13), 97 (24), 83 (29), 69 (35); Anal. Calcd. for C_38_H_29_N_7_OS (631.75): C, 72.25; H, 4.63; N, 15.52; S, 5.08; found: C, 72.23; H, 4.63; N, 15.51; S, 5.09.

*2-(5-(Furan-2-yl)-3-(p-tolyl)-4,5-dihydro-1H-pyrazol-1-yl)-4-phenylthiazole* (**16**). A mixture of 5-(furan-2-yl)-3-(*p*-tolyl)-4,5-dihydro-1*H*-pyrazole-1-carbothioamide (1.42 g, 5 mmol) and ω-bromo-acetophenone (0.99 g, 5 mmol) in ethanol (30 mL) was heated under reflux for 2 h to give *2-(5-(Furan-2-yl)-3-(p-tolyl)-4,5-dihydro-1H-pyrazol-1-yl)-4-phenylthiazole* (**15**) as a white precipitate which was washed with water and recrystallized from glacial acetic acid a white solid, yield (1.9 g, 71%), mp: 221 °C; IR (KBr, cm^−1^): 3100; 3041 (=C–H), 2943 (–C–H), 1717 (–C=O amide); ^1^H-NMR: δ 2.45 (s, 3H, 4-CH_3_C_6_H_4_), 3.77 (dd, 1H, pyrazoline-H), 3.93 (dd, 1H, pyrazoline-H), 6.34 (q, 1H, pyrazoline-H), 6.70–7.91 (m, 13H, Ar–H + 3furyl-H + 1thiazole H-5); MS (*m*/*z*): 385 (M+, 7%), 381 (3%), 378 (10%), 374 (23%), 369 (51%), 263 (13%), 262 (100%), 260 (12%), 223 (12%), 216 (20%), 215 (12%), 77 (33%), 76 (16%); Anal. Calcd. for C_23_H_19_N_5_O_2_S (385.48): C, 71.66; H, 4.97; N, 10.90; S, 8.32; found: C, 71.63; H, 4.98; N, 10.92; S, 8.32.

#### 4.1.5. (2-Phenylhydrazono)thiazol-4(5*H*)-one Derivatives **18a**–**e**

##### Method A

Equimolar amounts of the appropriate thioamides **13a**–**d**, **13f** and ethyl 2-chloro-2-(2-phenylhydrazono)acetate with triethylamine (5 mmol) in ethanol (25 mL) were refluxed for 2 h. The solid so formed was collected and crystallized from glacial acetic acid to afford 2-phenylhydrazono)thiazol-4(5*H*)-one **18a**–**e**, respectively.

##### Method B

Benzenediazonium chloride (5 mmol), which prepared from aniline (0.45 mL, 5 mmol), hydrochloric acid (6 N, 6 mL), and sodium nitrite (0.35 g, 5 mmol), was added dropwise with stirring to a cold solution of **19** (5 mmol) and sodium acetate trihydrate (1.3 g, 10 mmol) in ethanol (50 mL). The reaction mixture was stirred in ice bath for 3 h. The resulting solid was collected and crystallized to give a product identical in all aspects (mp, mixed mp, and spectra) with **18a**.

*(E)-2-(5-(Furan-2-yl)-3-(p-tolyl)-4,5-dihydro-1H-pyrazol-1-yl)-5-(2-phenylhydrazono)-thiazol-4(5H)-one* (**18a**). Yellow solid, yield (1.4 g, 67%), mp: 247–248 °C; IR (KBr, cm^−1^): 3432 (N–H), 2970; 2921 (–C–H), 1717 (–C=O amide); ^1^H-NMR: δ 2.39 (s, 3H, 4-CH_3_C_6_H_4_), 3.63 (dd, 1H, pyrazoline-H), 4.04 (dd, 1H, pyrazoline-H), 5.96 (q, 1H, pyrazoline-H), 6.44 (q, 1H, furyl-H), 6.54 (d, 1H, furyl-H), 6.9–7.78 (m, 10H, Ar–H + 1furyl-H), 10.4 (s, 1H, N–H); MS (*m*/*z*): 431 (M + 2, 1), 430 (M + 1, 29), 429 (M^+^, 100), 413 (15), 346 (15), 345 (40), 316 (10), 264 (23), 263 (11), 248 (10), 228 (58), 227 (25), 226 (16), 214 (44), 212 (12), 200 (12), 198 (11), 196 (16), 186 (14), 172 (11), 170 (13), 158 (14), 157 (10), 156 (13), 146 (17), 144 (18), 130 (16), 115 (10); Anal. Calcd. for C_23_H_19_N_5_O_2_S (429.49): C, 64.32; H, 4.46; N, 16.31; S, 7.47; found: C, 64.33; H, 4.46; N, 16.32; S, 7.44.

*(Z)-2-(3',5-di(Furan-2-yl)-1'-phenyl-3,4-dihydro-1'H,2H-[3,4'-bipyrazol]-2-yl)-5-(2-phenylhydrazono)-thiazol-4(5H)-one* (**18b**). Yellow solid, yield (1.7 g, 63%), mp: 284–285 °C; IR (KBr, cm^−1^): 3407 (N–H), 3044 (=C–H), 2852 (–C–H), 1635 (C=O); ^1^H-NMR: δ 3.08 (dd, 1H, pyrazoline-H), 3.90 (dd, 1H, pyrazoline-H), 6.19 (dd, 1H, pyrazoline-H), 6.66–7.90 (m, 17H, Ar–H + 1N–H + 6furyl-H), 8.09 (s, 1H, pyrazole-H-5); MS (*m*/*z*): 549 (M + 2, 1), 548 (M + 1, 6), 547 (M+, 16), 374 (100), 329 (7), 254 (19), 227 (61), 212 (17), 173 (77), 91 (9), 77 (7); Anal. Calcd. for C_29_H_21_N_7_O_3_S (547.59): C, 63.61; H, 3.87; N, 17.91; S, 5.86; found: C, 63.61; H, 3.88; N, 17.93; S, 5.80.

*(E)-2-(3'-(Furan-2-yl)-1'-phenyl-5-(p-tolyl)-3,4-dihydro-1'H,2H-[3,4'-bipyrazol]-2-yl)-5-(2-phenylhydrazono)-thiazol-4(5H)-one* (**18c**). Yellow solid, yield (1.5 g, 54%), mp: 245–247 °C; IR (KBr, cm^−1^): 3396 (N-H), 3140 (=C–H), 2990 (–C–H), 1715 (–C=O); ^1^H-NMR: δ 2.34 (s, 3H, 4-CH_3_C_6_H_4_), 3.18 (dd, 1H, pyrazoline-H), 3.90 (dd, 1H, pyrazoline-H), 6.17 (dd, 1H, pyrazoline-H), 6.65–8.08 (m, 19H, Ar–H + 3furyl-H + 1N–H + 1pyrazole-H-5); MS (*m*/*z*): 571 (M+, 2), 569 (4), 553 (10), 398 (12), 370 (15), 248 (51), 235 (100), 155 (12), 107 (10), 91 (27), 77 (2), 55 (9); Anal. Calcd. for C_32_H_25_N_7_O_2_S (571.65): C, 67.23; H, 4.41; N, 17.15; S, 5.61; found: C, 67.25; H, 4.41; N, 17.13; S, 5.60.

*(E)-2-(1'-Phenyl-3',5-di-p-tolyl-3,4-dihydro-1'H,2H-[3,4'-bipyrazol]-2-yl)-5-(2-phenylhydrazono)-thiazol-4(5H)-one* (**18d**). Yellow solid, yield (1.6 g, 55%), mp: 278–279 °C; IR (KBr, cm^−1^): 3431 (N–H), 3140 (=C–H), 2919 (–C–H), 1715 (–C=O); ^1^H-NMR: δ 2.34 (s, 3H, 4-CH_3_C_6_H_4_), 2.36 (s, 3H, CH_3_), 3.30 (dd, 1H, pyrazoline-H), 3.85 (dd, 1H, pyrazoline-H), 6.10 (dd, 1H, pyrazoline-H), 7.24–8.08 (m, 20H, 18Ar-H + 1N–H + pyrazole-H-5); MS (*m*/*z*): 595 (M+, 1), 578 (11), 554 (6), 397 (10), 340 (7), 279 (100), 263 (4), 236 (29), 193 (28), 91 (1); Anal. Calcd. for C_35_H_29_N_7_OS (595.72): C, 70.57; H, 4.91; N, 16.46; S, 5.38; found: C, 70.58; H, 4.92; N, 16.49; S, 5.32.

*(E)-2-(5-(Furan-2-yl)-1'-phenyl-3'-(p-tolyl)-3,4-dihydro-1'H,2H-[3,4'-bipyrazol]-2-yl)-5-(2-phenylhydrazono)-thiazol-4(5H)-one* (**18e**). Yellow solid, yield (2.1 g, 75%), mp: 294–296 °C; IR (KBr, cm^−1^): 3433 (N-H), 3137 (=C–H), 2921 (–C–H), 1708 (–C=O); ^1^H-NMR: δ 2.37 (s, 3H, 4-CH_3_C_6_H_4_), 3.40 (dd, 1H, pyrazoline-H), 4.10 (dd, 1H, pyrazoline-H), 5.90 (q, 1H, pyrazoline-H), 6.72 (q, 1H, furyl-H), 6.90–7.99 (m, 16H, Ar–H + 2furyl-H), 8.52 (s, 1H, pyrazole-H-5), 10.33(s, 1H, N-H); MS (*m*/*z*): 571 (M+, 2), 553 (10), 398 (12), 370 (15), 248 (51), 235 (100), 155 (12), 107 (10), 91 (27), 71 (4); Anal. Calcd. for C_32_H_25_N_7_O_2_S (571.65): C, 67.23; H, 4.41; N, 17.15; S, 5.61; found: C, 67.20; H, 4.41; N, 17.13; S, 5.64.

*2-(5-(Furan-2-yl)-3-(p-tolyl)-4,5-dihydro-1H-pyrazol-1-yl)-thiazol-5(4H)-one* (**19**) Equimolar amounts of 5-(furan-2-yl)-3-(p-tolyl)-4,5-dihydro-1*H*-pyrazole-1-carbothioamide (**13a**, 1.42 g, 5 mmol) and ethyl chloroacetate (0.61 g, 5 mmol) in ethanol (25 mL) were heated under reflux for 2 h, then allowed to cool at room temperature, the solid so formed was collected and recrystallized from dioxane to give 18 as a pale yellow solid, yield (1.2 g, 72%), mp: 244–245 °C; IR (KBr, cm^−1^): 3143 (=C–H aromatic), 3039 (=C–H), 2991 (–C–H), 1697 (C=O); ^1^H-NMR: δ 2.44 (s, 3H, 4-CH_3_C_6_H_4_), 3.61 (dd, 1H, pyrazoline-H), 3.92 (s, 2H, thiazole-H), 3.95 (dd, 1H, pyrazoline-H), 5.85 (q, 1H, pyrazoline-H), 6.42–7.76 (m, 7H, 4Ar-H + 3furyl-H); MS (*m*/*z*): 327 (M + 2, 1), 326 (M + 1, 10), 325 (M+, 50), 308 (47), 293 (100), 275 (51), 101 (35), 77 (40), 69 (67); Anal. Calcd. for C_17_H_15_N_3_O_2_S (325.38): C, 62.75; H, 4.65; N, 12.91; S, 9.85; found: C, 62.74; H, 4.67; N, 12.92; S, 9.81.

#### 4.1.6. General Procedure for the Synthesis of Compounds **20** and ***27***

Equimolar amounts of the appropriate pyrazole aldehyde **1a** or **2b** (10 mmol), *N*,*N*^`^-dimethylbarbituric acid (1.56 g, 10 mmol), thiourea (0.76 g, 10 mmol) and conc. hydrochloric acid (5 mL) in ethanol (25 mL) were heated under refluxed for 1 h. The reaction mixture was allowed to cool to room temperature and the precipitate was filtered and crystallized from dioxane to give compounds **20** and **27**, respectively.

*1,3-Dimethyl-5-(1-phenyl-3-(p-tolyl)-1H-pyrazol-4-yl)-7-thioxo-5,6,7,8-tetrahydro-pyrimido[4,5-d]pyrimidine-2,4(1H,3H)-dione* (**20**). Yellow solid, yield (4.3 g, 94%), mp: 267–268 °C; IR (KBr, cm^−1^): 3434 (N–H), 3175 (=C-H aromatic), 3019 (C=H), 2921 (–C–H), 1669 (C=O); ^1^H-NMR: δ 2.45 (s, 3H, 4-CH_3_C_6_H_4_), 3.40 (s, 3H, N–CH_3_), 3.44 (s, 3H, N–CH_3_), 7.27–7.93 (m, 11H, Ar-H + pyrimidine-H-5 + pyrazole-H-5), 8.62 (s, 1H, N–H), 9.88 (s, 1H, N–H); MS (*m*/*z*): 460 (M + 2, 2), 459 (M + 1, 12), 458 (M+, 36), 426 (73), 398 (33), 394 (28), 383 (52), 368 (38), 367 (100), 365 (16), 305 (10), 289 (34); Anal. Calcd. for C_24_H_22_N_6_O_2_S (458.54): C, 62.86; H, 4.84; N, 18.33; S, 6.99; found: C, 62.86; H, 4.85; N, 18.34; S, 6.98.

*5-(3-(Furan-2-yl)-1-phenyl-1H-pyrazol-4-yl)-1,3-dimethyl-7-thioxo-5,6,7,8-tetrahydropyrimido[4,5-d]pyrimidine-2,4(1H,3H)-dione* (**27**). Brown solid, yield (3.6 g, 85%), mp: 186–187 °C; IR (KBr, cm^−1^): 3439 (N-H), 3170 (=C–H), 2954 (–C–H), 1664 (C=O); ^1^H-NMR: δ 3.44 (s, 6H, 2N–CH_3_), 6.59–7.90 (m, 10H, ArH + 3furyl-H + pyrimidine-H+ pyrazole-H-5), 9.13 (s, 1H, N–H), 9.85 (s, 1H, N–H); MS (*m*/*z*): 434 (M+1, 3), 433 (M+, 5), 310 (100), 296 (51), 238 (49), 168 (28), 166 (34), 150 (17), 139 (59), 138 (42), 125 (74), 99 (11), 83 (14), 43 (22); Anal. Calcd. for C_21_H_18_N_6_O_3_S (434.47): C, 58.05; H, 4.18; N, 19.34; S, 7.38; found: C, 58.01; H, 4.19; N, 19.36; S, 7.38.

#### 4.1.7. General procedure for the Synthesis of Hexahydropyrimido[4,5-*d*][1,2,4]triazolo[4,3-*a*]pyrimidine Derivatives **26a**–**c** and **28a**,**d**

Equimolar amounts of **20** (2.29 g, 5 mmol) or **27** (2.17 g, 5 mmol) and the appropriate hydrazonyl halides **5a**–**d** (5 mmol) in ethanol (25 mL) containing 5 drops of triethylamine was heated under reflux for 20 h. The reaction mixture was evaporated under reduced pressure and the resulting solid was washed several times with water and ether. The precipitates formed, were filtered and recrystallized to give:

*Ethyl 7,9-dimethyl-6,8-dioxo-1-phenyl-5-(1-phenyl-3-(p-tolyl)-1H-pyrazol-4-yl)-1,5,6,7,8,9-hexahydropyrimido[4,5-d][1,2,4]triazolo[4,3-a]pyrimidine-3-carboxylate* (**26a**). White solid from glacial acetic acid, yield (2.4 g, 77%), mp: 250–251 °C; IR (KBr, cm^−1^): 3108 (=C–H aromatic), 3038 (=C–H), 2965; 2916 (–C–H), 1696 (C=O); ^1^H-NMR: δ 1.28 (t, 3H,–CH_2_CH_3_), 2.34 (s, 3H, 4-CH_3_C_6_H_4_), 2.82 (s, 3H, N–CH_3_), 2.86 (s, 3H, N–CH_3_), 4.30 (q, 2H,-OCH_2_CH_3_), 5.59 (s, 1H, pyrimidine-H), 6.88–7.7 (m, 14H, Ar–H), 7.77 (s, 1H, pyrazole-H-5); MS (*m*/*z*): 614 (M+, 9), 563 (6), 379 (10), 323 (54), 311 (30), 295 (100), 284 (11), 267 (19), 239 (4), 111 (12), 96 (22), 85 (18), 71 (26); Anal. Calcd. for C_34_H_30_N_8_O_4_ (614.65): C, 66.44; H, 4.92; N, 18.23; found: C, 66.43; H, 4.91; N, 18.21.

*3-Benzoyl-7,9-dimethyl-1-phenyl-5-(1-phenyl-3-(p-tolyl)-1H-pyrazol-4-yl)-7,9-dihydropyrimido[4,5-d][1,2,4]triazolo[4,3-a]pyrimidine-6,8(1H,5H)-dione* (**26b**). White solid from toluene, yield (1.6 g, 50%), mp: 211–213 °C; IR (KBr, cm^−1^): 3049 (=C–H), 2920 (–C–H), 1695 (–C=O); ^1^H-NMR: δ 2.43 (s, 3H, 4-CH_3_C_6_H_4_), 2.80 (s, 3H, N–CH_3_), 2.88 (s, 3H, N–CH_3_), 5.85 (s, 1H, pyrimidine-H), 6.86–7.68 (m, 19H, Ar-H), 8.32 (s, 1H, pyrazole-H-5); MS (*m*/*z*): 646 (M+, 3), 645 (6), 662 (5), 406 (13), 389 (54), 256 (12), 239 (14), 195 (11), 168 (13), 151 (100), 135 (29), 106 (17), 85 (30); Anal. Calcd. for C_38_H_30_N_8_O_3_ (646.70): C, 70.58; H, 4.68; N, 17.33; found: C, 70.56; H, 4.68; N, 17.34.

*7,9-Dimethyl-6,8-dioxo-N,1-diphenyl-5-(1-phenyl-3-(p-tolyl)-1H-pyrazol-4-yl)-1,5,6,7,8,9-hexahydropyrimido[4,5-d][1,2,4]triazolo[4,3-a]pyrimidine-3-carboxamide* (**26c**). White solid from glacial acetic acid, yield (2 g, 60%), mp: 258–259 °C; IR (KBr, cm^−1^): 3364 (N–H), 3136 (=C–H aromatic), 3054 (=C–H), 2921 (–C–H), 1690 (C=O); ^1^H-NMR: δ 2.42 (s, 3H, 4-CH_3_C_6_H_4_), 2.80 (s, 3H, N–CH_3_), 2.87 (s, 3H, N–CH_3_), 5.76 (s, 1H, pyrimidine-H), 6.86–7.68 (m, 19H, Ar-H), 7.78 (s, 1H, pyrazole-H-5), 8.55 (s, 1H, N–H); MS (*m*/*z*): 661 (M + 1, 3), 660 (M+, 7), 603 (9), 441 (11), 401 (1), 315 (29), 204 (100), 189 (46), 161 (12), 147 (56), 121 (3); Anal. Calcd. for C_38_H_31_N_9_O_3_ (661.71): C, 68.97; H, 4.72; N, 19.05; found: C, 68.93; H, 4.73; N, 19.07.

*Ethyl 5-(3-(furan-2-yl)-1-phenyl-1H-pyrazol-4-yl)-7,9-dimethyl-6,8-dioxo-1-phenyl-1,5,6,7,8,9-hexahydropyrimido[4,5-d][1,2,4]triazolo[4,3-a]pyrimidine-3-carboxylate* (**28a**). White solid from benzene, yield (2.2 g, 75%), mp: 249–251 °C; IR (KBr, cm^−1^): 3038 (=C–H), 2969 (–C–H), 1696 (C=O); ^1^H-NMR: δ 1.28 (t, 3H, –CH_2_CH_3_), 2.82 (s, 3H, N–CH_3_), 2.85 (s, 3H, N–CH_3_), 4.27 (q, 2H, –OCH_2_ CH_3_), 5.58 (s, 1H, pyrimidine-H), 6.87–7.69 (m, 13H, Ar–H + 3furyl-H), 7.75 (s, 1H, pyrazole-H-5); MS (*m*/*z*): 590 (M+, 1), 511 (2), 397 (10), 340 (7), 279 (100), 236 (29), 193 (28); Anal. Calcd. for C_31_H_26_N_8_O_5_ (590.59): C, 63.04; H, 4.44; N, 18.97; found: C, 63.01; H, 4.46; N, 18.91.

*5-(3-(Furan-2-yl)-1-phenyl-1H-pyrazol-4-yl)-7,9-dimethyl-6,8-dioxo-N,1-diphenyl-1,5,6,7,8,9-hexahydropyrimido[4,5-d][1,2,4]triazolo[4,3-a]pyrimidine-3-carboxamide* (**28b**). White solid from toluene, yield (1.7 g, 55%), mp: 145–146 °C; IR (KBr, cm^−1^): 3398 (N–H), 3130 (=C-H aromatic), 3028 (=C–H), 2949 (–C–H); ^1^H-NMR: δ 2.84 (s, 3H, N–CH_3_), 3.35 (s, 3H, N–CH_3_), 6.03 (s, 1H, pyrimidine-H), 6.53 (q, 1H, furyl-H), 6.91–7.67 (m, 17H, Ar–H + 2furyl-H), 8.73 (s, 1H, pyrazole-H-5), 8.54 (s, 1H, N–H); MS (*m*/*z*): 637 (M+, 7), 633 (17), 620 (24), 528 (11), 436 (25), 418 (34), 380 (24), 278 (18), 263 (66), 232 (34), 219 (100), 203 (57), 159 (89); Anal. Calcd. for C_35_H_27_N_9_O_4_ (637.65): C, 65.93; H, 4.27; N, 19.77; found: C, 65.90; H, 4.28; N, 19.73.

### 4.2. Antimicrobial Activity Assay

Chemical compounds under investigation were individually tested against a panel of Gram-positive and Gram-negative bacteria pathogens, and fungi. Antimicrobial tests were carried out using the agar well-diffusion method [46]. After the media had cooled and solidified, wells (6 mm in diameter) were made in the solidified agar, after that microbial inoculum was uniformly spread using sterile cotton swab on a sterile Petri dish containing nutrient agar (NA) medium or Sabouraud dextrose agar (SDA) media for bacteria and fungi, respectively. A 100 µL solution was prepared from 1 mL of DMSO by dissolving 1 mg of the compound. The inoculated plates were then incubated for 24 h at 37 °C for bacteria and yeast, 48 h at 28 °C for fungi. Negative controls were prepared using DMSO employed for dissolving the tested compound. Amphotericin B (1 mg/mL), ampicillin (1 mg/mL), and gentamicin (1 mg/mL) were used as standards for bacteria and fungi, respectively. After incubation, antimicrobial activity was evaluated by measuring the zone of inhibition against tested microorganisms. Antimicrobial activity was expressed as inhibition diameter zones in millimeters (mm). The experiment was carried out in triplicate and the average zones of inhibition ± S.D were calculated.

## 5. Conclusions

New series of novel functionalized 1,3,4-thiadiazoles, 1,3-thiazoles, and pyrimido[4,5-*d*][1,2,4]triazolo[4,3-*a*]pyrimidines containing pyrazole moieties were synthesized using hydrazonoyl halides as precursors and evaluated for their in vitro antibacterial, and antifungal activities. From the screening results, it can be seen *Aspergillus fumigatus* was susceptible to compounds **12b**, **13b**, **14b**, **15b**, and **15c** when compared to the amphotericin B standard. *Candida albicans* was susceptible to compounds **12b** and **13b** when compared to the amphotericin B standard*. Streptococcus pneumoniae* was susceptible to compounds **12b**, **13b**, and **14b** when compared to an ampicillin standard. *Bacillus subtilis* was susceptible to compounds **13b**, **14b**, and **15b** when compared to ampicillin standard. *Pseudomonas aeruginosa* was susceptible to compounds **12b**, **13b**, **15a**, and **26d** when compared to Gentamicin standard. *Escherichia coli* was susceptible to compounds **10a**, **12b**, **13b**, **14b**, **15f**, and **28a** when compared to the gentamicin standard.

## Figures and Tables

**Table 1:** Mean zone of inhibition beyond well diameter (6 mm) produced on a range of clinically pathogenic microorganisms using (5 mg/mL) concentration of tested samples.

Compound	*Aspergillus fumigatus* (Fungus)	*Candida albicans* (Fungus)	*Streptococcus pneumoniae (Gram* +ve Bact.)	*Bacillus subtilis (*Gram +ve *Bact.)*	*Pseudomonas aeruginosa (Gram* −ve Bact*.)*	*Escherichia coli* (*Gram* −ve Bact.)
**9a**	0	0	0	0	0	0
**9b**	15.7	0	16.2	19.8	0	16.3
**9c**	15.6	0	19.6	19.3	0	14.9
**10a**	16.3	0	18.9	21.3	0	19.9
**10b**	18.8	0	17.3	18.2	0	17.9
**10c**	18.6	0	20.6	20	0	15.9
**13b**	23.7	25.4	23.8	32.4	17.3	19.9
**13c**	14.6	16.9	10.2	9.8	0	11.2
**13f**	13.6	11.7	15.6	15.3	0	9.4
**14a**	15.9	15.1	17.3	13.3	11.9	11.6
**14b**	25.3	17.6	22.6	33.7	13.1	19.3
**14c**	10.3	11.3	8.3	10.9	0	8.8
**14e**	15.6	12.5	17.3	13.3	10.3	10.9
**14f**	0	0	12.6	11.2	0	9.6
**15a**	16.9	18.3	17.3	13.4	15.1	13.2
**15b**	22.3	16.5	19.5	30.8	12.3	17.6
**15c**	20.8	18.9	19.4	14.9	13.6	11.8
**15e**	15.4	12.9	19.6	13.9	9.7	8.9
**15f**	21.3	17.2	18.2	20.3	0	20.3
**18a**	17.1	11.1	13.9	14.2	11.7	10.3
**20**	15.4	14.8	10.9	12.9	11.3	11.6
**26a**	14.7	10.9	12.2	14.9	11.8	14.7
**26c**	13.9	15.6	17.3	20.6	12.5	14.8
**26d**	14.9	10.4	21.8	23.3	15.3	19.1
**27**	16.2	14.9	15.6	15.6	11.7	10.8
**28a**	16.3	13.4	17.5	20.8	0	18.9
**28b**	18.6	15.4	13.3	12.7	0	8.5
**Amphotericin B**	23.7	25.4	-	-	-	-
**Ampicillin**	-	-	23.8	32.4	-	-
**Gentamicin**	-	-	-	-	17.3	19.9

**Table 2:** Antimicrobial activity as MICS, (mg/mL) of tested samples against tested microorganisms.

Compound	*Aspergillus fumigatus* (Fungus)	*Candida albicans* (Fungus)	*Streptococcus pneumoniae* (Gram +ve Bact.)	*Bacillus subtilis* (Gram +ve Bact.)	*Pseudomonas aeruginosa* (Gram −ve Bact.)	*Escherichia coli* (Gram −ve Bact.)
**9d**	7.81	0	3.9	1.95	0	15.63
**10a**	31.25	0	3.9	3.9	0	3.9
**10b**	7.81	0	31.25	7.81	0	15.63
**10c**	3.9	0	3.9	3.9	0	31.25
**13b**	12.5	62.5	3.9	3.9	12.5	15.63
**14b**	25	12.5	15.63	7.95	50	31.25
**15f**	12.5	62.5	3.9	1.95	25	15.63
**28a**	25	50	62.5	62.5	0	25
**Amphotericin B**	0.49	0.49	-	-	-	-
**Ampicillin**	-	-	0.49	0.24	-	-
**Gentamicin**	-	-	-	-	15.63	3.9

**Table 3:** Antimicrobial activity as half maximal inhibitory concentration (IC_50_) (mg/mL) of tested samples against tested microorganisms.

Compound	*Aspergillus fumigatus* (Fungus)	*Syncephalastrum racemosum* (Fungus)	*Geotrichum candidum* (Fungus)	*Candida albicans* (Fungus)	*Streptococcus pneumoniae* (Gram +ve Bact.)	*Bacillus subtillis* (Gram +ve Bact.)	*Pseudomonas aeruginosa* (Gram −ve Bact.)	*Escherichia coli* (Gram −ve Bact.)
**9d**	43.21	36.28	18.24	0	17.52	15.63	0	27.34
**10a**	64.31	34.28	31.17	0	31.25	18.24	0	31.56
**10b**	42.63	33.42	19.63	0	76.34	37.25	0	28.37
**10c**	35.24	27.58	24.63	0	25.12	22.41	0	34.25
**Amphotericin B**	11.24	16.84	9.32	12.68	-	-	-	-
**Ampicillin**	-	-	-	-	10.58	5.29	-	-
**Gentamicin**	-	-	-	-	-	-	17.96	16.24
